# Antiretroviral (ARV) Drug Resistance and HIV-1 Subtypes among Injecting Drug Users in the Coastal Region of Kenya

**DOI:** 10.1155/2022/3217749

**Published:** 2022-02-10

**Authors:** Gabriel O. Ng'ong'a, George Ayodo, Fanuel Kawaka, Veronicah Knight, Musa Ngayo, Raphael M. Lwembe

**Affiliations:** ^1^Department of Public Health, Jaramogi Oginga Odinga University of Science and Technology (JOOUST), P.O. Box 210–40601, Bondo, Kenya; ^3^Department of Biological Sciences, Jaramogi Oginga Odinga University of Science and Technology (JOOUST), P.O. Box 210 – 40601, Bondo, Kenya; ^2^Center for Viral Research, Kenya Medical Research Institute (KEMRI), P.O. Box 54840-00200, Nairobi, Kenya

## Abstract

HIV-1 genetic diversity results into the development of widespread drug-resistant mutations (DRMs) for the first-line retroviral therapy. Nevertheless, few studies have investigated the relationship between DRMs and HIV-1 subtypes among HIV-positive injecting drug users (IDUs). This study therefore determined the association between HIV-1 genotypes and DRMs among the 200 IDUs. Stanford HIV Drug Resistance Database was used to interpret DRMs. The five HIV-1 genotypes circulating among the IDUs were A_1_ (25 (53.2%)), A_2_ (2 (4.3%)), B (2 (4.3%)), C (9 (19.1%)), and D (9 (19.1%)). The proportions of DRMs were A_1_ (12 (52.2%)), A_2_ (1 (4.3%)), B (0 (0.0%)), C (5 (21.7%)), and D (5 (21.7%)). Due to the large proportion of drug resistance across all HIV-1 subtypes, surveillance and behavioral studies need to be explored as IDUs may be spreading the drug resistance to the general population. In addition, further characterization of DRMs including all the relevant clinical parameters among the larger population of IDUs is critical for effective drug resistance surveillance.

## 1. Introduction

The introduction of highly active antiretroviral therapy (HAART) in 1996 has resulted in improved treatment outcome and survival rate in human immunodeficiency virus-1 (HIV-1) infected patients [1–3]. The success of these drug regimens is being challenged by the emergence of drug-resistant mutations [1]. Indeed, transmitted drug resistance generally leads to a delay in virologic suppression [4, 5] and results in an increased risk of treatment failure [6]. Drug resistance testing and monitoring of HIV subtypes can improve treatment outcomes in infected individuals [7, 8]. However, limited studies have been done on the high-risk group such as injecting drug users (IDUs). These mutant variants have become increasingly widespread, in drug-treated and untreated individuals infected with HIV, and have compromised the therapeutic options of drug-naïve infected people [6, 9].

Studies in Kenya have reported the occurrence of HIV drug resistance upon ART failure in Mombasa [10], Mombasa and Nairobi as part of a multisite African study [11], and Burnt Forest, a rural Academic Model Providing Access to Healthcare (AMPATH) clinic [12]. HIV-1 infection is highly diverse with the circulation of subtypes A (50–80%), D (10–20%), and C (5–15%) and multiple recombinants (10–20%) [13, 14]. Extensive genetic heterogeneity is driven by several factors, such as the lack of proofreading ability of the reverse transcriptase (RT) [15], the rapid turnover of HIV-1 in vivo [16], host-selective immune pressures [17], leading to drug resistance selection pressure, and recombination events during replication [18]. HIV type 1 is divided into groups M, N, O, and P, more than 90% of HIV infections are derived from HIV-1 group M, and the rest are minor groups [19, 20]. The M group is subdivided further into clades, called subtypes, which are also given letters ranging from A to K. Subtype A has been subdivided into A_1_, A_2_, A_3_, A_4_, A_5_, and A_6_, while subtype F has been subdivided into F_1_ and F_2_ [21]. This study therefore assessed the relationship between HIV-1-circulating genotypes and drug-resistant mutations among IDUs.

## 2. Methods

### 2.1. Study Setting

A cross-sectional survey was conducted among HIV-positive injecting drug users in Malindi Sub-County, coastal region of Kenya. The study enrolled 200 IDUs who consented and fulfilled eligibility criteria of being HIV infected, 18 years old and above, actively injecting drugs for the past six months, and were able to respond to structured questionnaires during interviews.

### 2.2. Participant Recruitment and Administration of Questionnaires

Snowball sampling was adopted since IDUs are hard to reach the population. A set of initial participants referred to as “seed” for an expanding chain of referrals. All the participants were provided with written informed consent, and only those who consented were recruited into the study. Each participant was assigned a confidential identification number. The reliability of the questionnaire was pretested on 20 respondents (10%) and revised based on their feedbacks. The structured questionnaires were then administered by a qualified counselor in a private room using face-to-face interviews for approximately 10 minutes. Sociodemographic information was also collected during the interviews.

### 2.3. Ethical Consideration

This study sought ethical approval from the Scientific Ethical Review Unit (SERU)—KEMRI (SSC no. 1438). Furthermore, permission was also sought from Jaramogi Oginga Odinga University of Science and Technology (JOOUST). Voluntary and written informed consent was obtained from the study participant before being allowed to take part in this study. Furthermore, the data collected from this study were confidential and only used for the purpose explained in the consent forms. Participation in this study presented no life-threatening risks.

### 2.4. Sample Collection

Blood samples were collected from HIV-positive IDUs with and without prior exposure to first-line antiretroviral therapy for HIV-1 subtypes and drug-resistant mutation analysis. About 5 ml of whole blood was drawn from each participant, separated into plasma, and transported to Kenya Medical Research Institute (KEMRI) under the cold chain for serological HIV-1 testing and molecular analysis. HIV colloidal gold, a rapid test for antibody to HIV, was used according to guidelines by the Ministry of Health for adult HIV testing [22, 23]. Whole blood was spun at 3,000 rpm for 3 minutes, and plasma aspirated aseptically and stored at −80°C for subsequent RNA extraction.

### 2.5. RNA Extraction and Genotyping

RNA was extracted from 140 *µ*l of plasma using a QIAmp viral RNA kit according to the manufacturer's instructions (Qiagen Inc., USA). A nested polymerase chain reaction (PCR) was performed using AmpliTaq Gold (Roche Molecular Systems, Branchburg, NJ) [24]. PCR products of correct size were confirmed by agarose gel electrophoresis, purified, and sequenced by dideoxynucleoside-based analysis using a BigDye terminator kit (Applied Biosystems) and ABI Prism 3100 equipment (Applied Biosystems, Foster City, US) [25].

### 2.6. Drug-Resistant Mutation Analysis

HIV drug resistance was defined as the presence of HIV mutations associated with impaired drug susceptibility. A nested PCR was performed using AmpliTaq Gold (Roche Molecular Systems, Branchburg, NJ) in the first round; HIV-1 pol gene was amplified using primers (RT18: 5′ GGAAACCAAAAATGATAGGGGGAATTGGAGG3′) and master mix consisting of 5 *μ*l H_2_O, 12.5 *μ*l 2X reaction mix, 1 *μ*l primer RT18, 1 *μ*l primer RT21, 1 *μ*l Platinum Taq, and 5 *μ*l RNA template and RT21 (5′ CTGTATTTCTGCTATTAAGTCTTTTGATGGG 3′). The second-round amplification includes primers (RT1: 5′CCAAAAGTTAAATGGCCATTGACAGA3′ and RT4: 5′AGTTCATAACCCATCCAAAG 3′) and master mix consisting of 27.7 *μ*l H_2_O, 5 *μ*l 10 × buffer, 5 *μ*l 25 mM MgCl_2_, 5 *μ*l dNTP 8 mM, 2.5 *μ*l primer RT1, 2.5 *μ*l primer RT4, 0.3 *μ*l Taq, and 2 *μ*l 1st round template. The PCR amplification was confirmed by visualization with ethidium bromide staining of agarose gel. The PCR-positive samples were cleaned off excess primers and nucleotides in a single step using ExoSAP-IT^™^ PCR technology according to manufacturer's instructions. Sequencing was achieved by dideoxynucleoside-based analysis using a BigDye terminator kit (Applied Biosystems) and ABI Prism 3300 equipment (Applied Biosystems, Foster City, US). Generated nucleotide sequences were edited using Sequencher^®^ 5.4.1 user 2015, Gene Codes Corporation Inc.

The identification and interpretation of drug-resistant mutations were done using the Stanford University and International AIDS Society, USA (http://hivdb.stanford.edu).

### 2.7. Statistical Methods

Chi-square (*χ*^2^) tests were done to establish the relationship between HIV-1 subtypes and drug resistance using Stata software version 13.0. Statistical significance was established at the 95% confidence limit within a marginal error of 0.05. Demographic data were analyzed using descriptive statistics; frequency (*n*) and percentage occurrence (%) of variables were generated using cross-tabulations.

## 3. Results

### 3.1. Sociodemographic Characteristics of Study Participants

This study involved 200 injecting drug users with 120 (60%) males and 80 (40%) females. 55% (*n* = 111) of the study participants were born in Malindi, 38% (*n* = 76) were from Kilifi, while 99% (*n* = 198) of the study participants were self-employed. A high proportion of this study population was unmarried (70.5%, *n* = 141), and 85% (*n* = 171) had a primary level of education. 70.5% (*n* = 141) were single with only 23.5% married (*n* = 47); the rest were divorcees (6.0%, *n* = 12) (Table 1).

The mean age of the study population was 33.36 years with a range of 19 to 82 years. Out of the extracted 200 RNA samples, 79 DNA samples were amplified, and 47 samples were successfully sequenced. The drug-resistant mutations were observed on 23 out of the 47 samples (48.9%).

### 3.2. HIV-1 Genotypes Circulating among the IDUs

**Figure 1:**
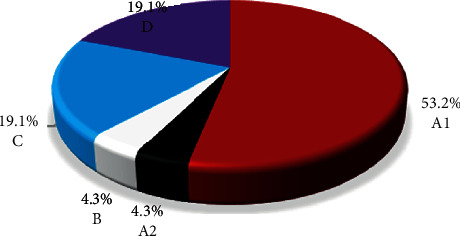
The proportion of HIV-1 subtypes.

As shown in Figure 1, a total of 5 HIV-1 subtypes, namely, A_1_, A_2_, B, C, and D, were observed. The HIV-1 subtype A_1_ was the most abundant (25 (53.2%)). Both subtypes C and D had a similar frequency of 19.1% compared to A_2_ and B in which both were 4.3%.

### 3.3. Drug-Resistant Mutations (DRMs) among Different HIV-1 Subtypes

Of the 47 study participants, 23 (48.9%) had the drug resistance as shown in Table 2. HIV-1 subtype A_1_ had the highest proportion of drug-resistant mutations (52.2%, *n* = 12), followed by both C and D which had 5 (21.7%) mutations. However, there was no significant association between the HIV-1 genotype and drug-resistant mutations (*χ*^2^ = 2.9752; *P* = 0.704). Other HIV-1 subtypes such as A_2_ and B had very low frequency of drug-resistant mutations.

E138A mutant genes had a high frequency of occurrence (3 times) in both NRTIs and NNRTIs. However, K103N mutations were more implicated in NNRTIs (4 times) than in NRTIs (once), while the individuals taking NNRTIs had also high chances of developing K103N, K219KN, and Y181C mutations (Table 3).

All the NRTIs exhibited the following mutations: M41ML, D67N, K70R, M184V, K219Q, V106A, L74LV, Y181YC, G190GA, K70KN, K70R, V75VI, M184LV, and K219HQ. It was also observed that all the NNRTIs manifested A98G, V179T, V106I, V179IL, and G190AS mutations (Table 4). These results further showed that HIV-1 subtype B exhibited no drug-resistant mutation.

It was observed that 34.8% (*n* = 8) of the 23 samples that exhibited major resistant mutations were ART-naïve individuals; they were drug-resistant de novo IDUs. These individuals were still on Septrin at the time of sample collection. Among the drug-naïve participants, it was established that HIV-1 subtype A_1_ exhibited most of mutant genes affecting both NRTIs (K70KN, D67N, K70R, V75VI, M184LV, K219HQ, Y115YF, K65KE, and L74L^*∗*^W) and NNRTs (V106VI, V179IL, and G190AS). NTRIs were observed to be the most resisted group of ART in this study. Subtype B had no mutant gene among the drug-naïve IDUs (Table 5).

Among ART-experienced IDUs, this study established 65.2% (*n* = 15) samples that showed major resistant mutations. It was revealed that those who had been on ART between 1 and 5 years had more mutations than those less than one year and also above five years. It was also observed that HIV-1 subtype A_1_ had the most abundant drug-resistant mutations. Mutant gene E138A was commonly observed in subtype C (Table 6).

## 4. Discussion

The study shows five HIV-1 genotypes circulating with the high proportion of drug-resistant mutations observed in HIV-1-A_1_ subtype among the IDUs. As much as our study shows a number of drug-resistant mutations in HIV-1-A_2_, a study conducted by Songok et al. [26] indicated that this subtype is rare in Kenya. Our study has also shown the presence of HIV-1 subtype B among the IDUs that had previously been reported to be predominantly found in the USA, Europe, Australia, Thailand, and Brazil [27, 28].

Moreover, earlier studies documented that HIV-1 subtypes are not randomly distributed among the globe and show distinct geographical distribution [29]. Subtypes A and D are the most dominant in Africa; subtype B in the USA, Europe, Australia, Thailand, and Brazil; subtype C in South Africa, Ethiopia, and India; F in some regions of Central Africa and Eastern Europe; and HIV-1-circulating recombinant form consisting of CRF01_AE in Southeast Asia [27]. The findings suggest a possible importation of rare subtypes, and this can be attributed to the commercial sex that is flourishing in the coastal region of Kenya. A study done in Kisumu, Kenya, reported that HIV-1 subtype was predominantly A (63%), followed by D (15%), C (3%), and G (1%) [30]. These results are in agreement with the findings from another study which reported A_1_ (41/65, 63.1%), C (7/65, 10.8%), D (16/65, 24.6%), and G (1/65, 1.5%) [31]. However, contrary results were reported in Uganda which established HIV-1 seroconverts infected with subtype A (15%) and D (59%), suggesting predominance of subtype D [32]. In Brazil, among 69 IDUs' samples amplified, 52 (75%) were identified as HIV-1 subtype B, 15 (22%) as subtype C, and 2 (3%) as subtype F (de Martínez et al. [32]). Kiwanuka et al. [33] reported that, globally, subtype C is the most successful of the HIV-1M lineages and accounts for >50% of infections, whereas subtypes A and B each account for over 10% of worldwide HIV infections. Subtypes D and G, CRF01_AE, and CRF02_AG account for only between 2% and 6% each. The difference in the proportions of HIV-1 subtypes suggests that it may be a driver of HIV-1-resistant mutation distribution in the population [34]. It is interesting that one HIV subtype can exhibit regional predominance. Perhaps, this subtype A has evolved to be more virulent than other strains due to improved replication fitness which may explain why it is more abundant.

Our study further shows that HIV-1 subtype A had significantly higher drug resistance followed by both C and D mutations. The finding is in agreement with other studies that reported having either HIV-1 subtype A or D is not associated with the acquisition of drug-resistant mutations [35]. However, M184V/I was significantly more common in subtype A as compared to subtype D. The absence of resistance mutation in subtype B is in contrast with other results [36] which established that the distributions of M184V/I were significantly associated with subtype B.

In the recent past, antiretroviral drugs were developed and efficacy tested with HIV-1 subtype B, and clinical effectiveness and pattern of drug resistance among subtype-B-infected individuals were established (Chaplin B. et al., 2011). Genetic differences between subtypes might impact the drug resistance pathways (Ode H. et al., 2007). It has been demonstrated that the mutational pathway to drug-resistant mutations to NRTI drugs may vary among different HIV-1 subtypes. However, the mechanisms and reason as to why this happens are yet to be fully assessed (Dumans A. T. et al., 2009). This could explain partly the absence of drug-resistant mutations in the HIV-1 B subtype in Kenya.

There was frequent occurrence of A62AV mutant genes in NRTIs followed by E138A mutation and A98G mutant genes in NNRTIs followed by K103N. These observations suggest that individuals on NRTIs are highly likely to develop A62AV and E138A, while those on NNRTIs are more likely to develop A98G and K103N mutant genes. Similarly, a study in Ghana among women with a history of prophylaxis recognized K103N and A98G as the dominant NNRTI mutations [36]. Furthermore, NNRTI mutations observed among drug-inexperienced individuals were K103N, V106A, and E138A with one minor drug resistance-associated mutation as A98G [36, 37]. Furthermore, HIV-1 subtype A exhibited most of mutant genes among drug-naïve participants affecting both NRTIs (K70KN, D67N, K70R, V75VI, M184LV, K219HQ, Y115YF, K65KE, and L74L^*∗*^W) and NNRTs (V106VI, V179IL, and G190AS). These findings show that NTRIs were the most resistant type of ART. However, studies in China revealed high percentages of NRTI (M184I/V) and NNRTI (K103N/S and Y181C/I) mutations in subtype B. High percentages of M184I/V (26.3%) and K103N/S (39.5%) were found in subtype B strains in ART-naïve individuals [38–40].

We have observed DRMs among the naïve and those using the drugs, and we have also observed more DRMs among those who have used ART for a long time. These suggest that the DRMs in ART-naïve IDUs could be due to acquisition of these mutations from patients failing treatment with resistant strains, prior exposure to ART, or undisclosed ART. This observation is in agreement with a recent study by Barik et al, (2021). Characterization of drug resistance mutations in RT gene of HIV-1 subtype C-infected individuals revealed that mutation M184V (63.15%) associated with lamivudine and abacavir and K103N (36.84%) identified in first-line ART failure in patients could be due to acquisition of these mutations from those failing treatment with resistant strains, prior exposures to ART, or undisclosed ART (41). This study is among the few that has investigated antiretroviral drug-resistant mutations and HIV-1 subtypes among hard-to-reach population of injecting drug users. However, snowball sampling approach used in this study is not representative and is prone to biases. Also, due to failure to consider the clinical parameters such as CD4^+^ cells and viral load, some samples failed to amplify. This is perhaps due to low copies of the virus that could not be detected by the nested RT-PCR. We however note that as much as the failure to amplify reduced the sample size, the findings are consistent with other studies.

## 5. Conclusion

All the HIV-1 subtypes have the drug-resistant mutations except subtype B. With drug-resistant mutations across all the HIV subtypes, there is a need to enhance the surveillance and more behavioral studies as IDUs may act as a source of drug resistance to the general population.

## Figures and Tables

**Table 1:** Demographic information of the study participants.

	Frequency (*n*)	Percentage
Gender		
Male	120	60
Female	80	40
Age (years)		
18–28	58	29
29–39	97	48.5
40–50	42	21
51 and above	3	1.5
Level of education		
Illiterate	0	0
Primary	170	85
Secondary	30	15
Tertiary	0	0
Marital status		
Single	141	70.5
Married	47	23.5
Divorced	12	6.0
Family type		
No family	144	72
Monogamous	45	22.5
Polygamous	11	5.5
Total	200	100

**Table 2:** Proportion of drug-resistant genotypes.

HIV-1 subtypes	No. of subtypes (%)	Sample with MDR (*n* (%))
A_1_	25 (53.2)	12 (52.2)
A_2_	2 (4.3)	1 (4.3)
B	2 (4.3)	0 (0.0)
C	9 (19.1)	5 (21.7)
D	9 (19.1)	5 (21.7)

**Table 3:** Mutations affecting both NRTI and NNRTI.

Mutation type	NRTI (f)	NNRTI (f)
E138A	3	3
K103N	1	4
K219KE	1	1
K219KN	1	3
K238KIN	1	1
K65KE	1	1
K70KN	1	1
K70KR	1	1
L100LF	1	1
T215TIN	1	1
Y115YF	1	2
Y181C	2	3

**Table 4:** Drug-resistant mutations circulating among HIV-1 subtypes.

HIV-1 subtypes	Antiretroviral	Mutations
A_1_	AZT	T215TIN, K70R, K219Q/E
	EFV	L100LF, Y181C, G190S
	NVP	K103N, Y181C, G190S, K101E,
	ABC	M184IV, K70KN, K70KR, Y115YF, K65KE, L74L^*∗*^W, D67N
	3TC	M184IV, M184V, L210^*∗*^W
	d4T	D67N, K219Q/E
	FTC	K103N, K219KE, Y115YF, M184V, L210^*∗*^W
	ddI	D67N
	TDF	D67N
	DOR	Y181C
	All NRTIs	M41ML, D67N, K70R, M184V, K219Q, V106A, L74LV, Y181YC, G190GA, K70KN, K70R, V75VI, M184LV, K219HQ, D67N
	All NNRTIs	A98G, V179T, V106I, V106VI, V179IL, G190AS
A_2_	ABC	K219KN
	FTC	K219KN
	3TC	K219KN, EFV, ETR, NVP E138A, L234LI, K238KIN, Y188L
	DOR	Y188L
B	Nil	
C	ETR	E138A
	RPV	E138A, K101E
D	AZT	T215TN
	EFV	K238N
	NVP	K238N
	DOR	L234L

**Table 5:** HIV-1 subtypes and the associated mutations among drug-naive IDUs.

HIV-1 subtypes	NRTI-associated mutation types	NNRTI-associated mutation types
A_1_	K70KN, D67N, K70R, V75VI, M184LV, K219HQ, Y115YF, K65KE, L74L^*∗*^W	V106VI, V179IL, G190AS
A_2_	M184V	Y188L
B	0	0
C	A62AV, K219KN	E138A, L234LI, K238KIN
D	T215TN	K238N

**Table 6:** Duration on ART and drug-resistant mutations among HIV-1 subtypes.

ART treatment duration	ART	HIV-1 subtypes	DRMs
<1 year	AZT/3TC/NVP	C	A62AV
	AZT/3TC/NVP	A_1_	L100LF, K103N

1–5 years	AZT/3TC/EFV	C	E138A
	AZT/3TC/NVP	C	E138A
	AZT/NVP/3TC/CPT	A_1_	M41ML, D67N, K70R, M184V, K219Q, G190A
	AZT/3TC/NVP	A_1_	G190A
	AZT/3TC/NVP	A_1_	M184V, L210^*∗*^W, A98G, Y181C
	TDF/3TC/EFV	A_1_	K70KR, T215TIN, K219KE
	TDF/3TC/EFV	D	L234LI
	TDF/3TC/EFV	C	E138A
	AZT, NVP, 3TC, CPT	A_1_	M41ML, D67N, K70R, M184V, K219Q, G190A
	NVP, AZT, 3TC, SPT	A_1_	M184V, A98G, K101E, V106VI, V179T, Y181C, G190S

>5 years	NVP, LUM, AZT, SPT	A_1_	M184IV, K103N, Y181C
	NVP, AZT, 3TC, SPT	D	K219N, L100LF, Y181F
	NVP, LUM, AZT, SPT	A_1_	L74LV, Y181YC, G190GA

## Data Availability

All data generated and analyzed are included within this research article.

## References

[1] Bure D., Makhdoomi M., Lodha R. (2015). Mutations in the reverse transcriptase and protease genes of human immunodeficiency virus-1 from antiretroviral naïve and treated pediatric patients. *Viruses*.

[2] Gatanaga H., Ibe S., Matsuda M. (2007). Drug-resistant HIV-1 prevalence in patients newly diagnosed with HIV/AIDS in Japan☆. *Antiviral Research*.

[3] Tang J. W., Pillay D. (2004). Transmission of HIV-1 drug resistance. *Journal of Clinical Virology*.

[4] Shubber Z., Mills E. J., Nachega J. B. (2016). Patient-reported barriers to adherence to antiretroviral therapy: a systematic review and meta-analysis. *PLoS Medicine*.

[5] Kuritzkes D. R., Lalama C. M., Ribaudo H. J. (2008). Preexisting resistance to nonnucleoside reverse‐transcriptase inhibitors predicts virologic failure of an efavirenz‐based regimen in treatment‐naive HIV‐1-Infected subjects. *Journal of Infectious Diseases*.

[6] Memarnejadian A., Menbari S., Mansouri S. A. (2015). Transmitted drug resistance mutations in antiretroviral-naïve injection drug users with chronic HIV-1 infection in Iran. *PLoS One*.

[7] Nasir I. A., Emeribe A. U., Ojeamiren I., Aderinsayo Adekola H. (2017). Human immunodeficiency virus resistance testing technologies and their applicability in resource-limited settings of Africa. *Infectious diseases*.

[8] Hirsch M. S., Günthard H. F., Schapiro J. M. (2008). Antiretroviral drug resistance testing in adult HIV‐1 infection: 2008 recommendations of an international AIDS society-USA panel. *Clinical Infectious Diseases*.

[9] Re M. C., Monari P., Bon I., Borderi M., Chiodo F. (2004). Conflicting interpretations of the prevalence of mutations associated with drug resistance in antiviral naïve HIV-1 patients with acute and chronic infection. *International Journal of Antimicrobial Agents*.

[10] Hassan A. S., Nabwera H. M., Mwaringa S. M. (2014). HIV-1 virologic failure and acquired drug resistance among first-line antiretroviral experienced adults at a rural HIV clinic in coastal Kenya: a cross-sectional study. *AIDS Research and Therapy*.

[11] Osman S., Lihana R. W., Kibaya R. M. (2013). Diversity of HIV type 1 and drug resistance mutations among injecting drug users in Kenya. *AIDS Research and Human Retroviruses*.

[12] Lihana R. W., Khamadi S. A., Lubano K. (2009). HIV type 1 subtype diversity and drug resistance among HIV type 1-infected Kenyan patients initiating antiretroviral therapy. *AIDS Research and Human Retroviruses*.

[13] Wang Z., Zhang M., Zhang R. (2019). Diversity of HIV-1 genotypes and high prevalence of pretreatment drug resistance in newly diagnosed HIV-infected patients in Shanghai, China. *BMC Infectious Diseases*.

[14] Kantor R., DeLong A., Balamane M. (2014). HIV diversity and drug resistance from plasma and non-plasma analytes in a large treatment programme in western Kenya. *Journal of the International AIDS Society*.

[15] Ciccozzi M., Lo Presti A., Cenci A. (2011). May phylogenetic analysis support epidemiological investigation in identifying the source of HIV infection?. *AIDS Research and Human Retroviruses*.

[16] Zhu W., Jiao Y., Lei R. (2011). Rapid turnover of 2-LTR HIV-1 DNA during early stage of highly active antiretroviral therapy. *PLoS One*.

[17] Currenti J., Chopra A., John M. (2019). Deep sequence analysis of HIV adaptation following vertical transmission reveals the impact of immune pressure on the evolution of HIV. *PLoS Pathogens*.

[18] González-Candelas F., Patiño-Galindo J., Valiente-Mullor C. (2018). Population genomics of human viruses. *Population Genomics: Microorganisms*.

[19] Sousa J. D., Müller V., Vandamme A.-M. (2017). The epidemic emergence of HIV: what novel enabling factors were involved?. *Future Virology*.

[20] Dhar D. V., Amit P., Kumar M. S. (2012). In-silico identification of new genes in HIV-1 by ORF prediction method. *International Research Journal of Biological Sciences*.

[21] Lihana R. W., Ssemwanga D., Abimiku A., Ndembi N. (2012). Update on HIV-1 diversity in Africa: a decade in review. *AIDS Reviews*.

[22] Mophs (2010). *National Guidelines for HIV Testing and Counselling in Kenya*.

[23] Mo H. (2012). *Guidelines for Antiretroviral Therapy in Kenya 2011*.

[24] Bessong P. O., Mphahlele J., Choge I. A. (2006). Resistance mutational analysis of HIV type 1 subtype C among rural South African drug-naive patients prior to large-scale availability of antiretrovirals. *AIDS Research and Human Retroviruses*.

[25] Macharia V. M., Ngugi C., Lihana R., Ngayo M. O. (2016). Transmitted HIV-1 drug resistance and the role of herpes simplex virus-2 coinfection among fishermen along the shores of Lake Victoria, Kisumu, Kenya. *Journal of HIV & Retro Virus*.

[26] Songok E. M., Lihana R. W., Kiptoo M. K. (2003). Identification ofenvCRF-10 among HIV variants circulating in rural western Kenya. *AIDS Research and Human Retroviruses*.

[27] Kandathil A. J., Ramalingam S., Kannangai R., David S., Sridharan G (2005). Molecular epidemiology of HIV. *Indian Journal of Medical Research*.

[28] Kato S., Saito Y., Tanaka R. (2003). Differential prevalence of HIV type 1 subtype B and CRF01_AE among different sexual transmission groups in Tokyo, Japan, as revealed by subtype-specific PCR. *AIDS Research and Human Retroviruses*.

[29] Kuiken C., Thakallapalli R., Eskild A., de Ronde A. (2000). Genetic analysis reveals epidemiologic patterns in the spread of human immunodeficiency virus. *American Journal of Epidemiology*.

[30] Otecko N., Inzaule S., Odhiambo C. (2016). Viral and host characteristics of recent and established HIV-1 infections in Kisumu based on a multiassay approach. *Scientific Reports*.

[31] Shao Y., Williamson C. (2012). The HIV-1 epidemic: low- to middle-income countries. *Cold Spring Harbor perspectives in medicine*.

[32] de Martínez A. M. B., Barbosa E. F., Ferreira P. C. P., Cardoso F. A., Silveira J. (2002). Molecular epidemiology of HIV-1 in rio grande, RS, Brazil. *Revista da Sociedade Brasileira de Medicina Tropical*.

[33] Kiwanuka N., Laeyendecker O., Robb M. (2008). Effect of human immunodeficiency virus type 1 (HIV‐1) subtype on disease progression in persons from rakai, Uganda, with incident HIV‐1 infection. *Journal of Infectious Diseases*.

[34] Gartner M. J., Roche M., Churchill M. J., Gorry P. R., Flynn J. K. (2020). Understanding the mechanisms driving the spread of subtype C HIV-1. *EBioMedicine*.

[35] Lu X., Zhao H., Zhang Y. (2017). HIV-1 drug-resistant mutations and related risk factors among HIV-1-positive individuals experiencing treatment failure in Hebei Province, China. *AIDS Research and Therapy*.

[36] Chaplin B., Eisen G., Idoko J. (2011). Impact of HIV type 1 subtype on drug resistance mutations in Nigerian patients failing first-line therapy. *AIDS Research and Human Retroviruses*.

[37] Martin-Odoom A., Brown C. A., Odoom J. K., Bonney E. Y., Ntim N. A. A. (2018). Emergence of HIV-1 drug resistance mutations in mothers on treatment with a history of prophylaxis in Ghana. *Virology Journal*.

[38] Martin-Odoom A., Adiku T., Delgado E., Lartey M., Ampofo W. K. (2017). Occurrence of transmitted HIV-1 drug resistance among Drug-naïve pregnant women in selected HIV-care centres in Ghana. *Ghana Medical Journal*.

[39] Zuo L., Liu K., Liu H. (2020). Trend of HIV-1 drug resistance in China: a systematic review and meta-analysis of data accumulated over 17 years (2001-2017). *EClinicalMedicine*.

[40] Onywera H., Maman D., Inzaule S. (2017). Surveillance of HIV-1 pol transmitted drug resistance in acutely and recently infected antiretroviral drug-naïve persons in rural western Kenya. *PLoS One*.

